# Androgen and estrogen sensitivity of bird song: a comparative view on gene regulatory levels

**DOI:** 10.1007/s00359-017-1236-y

**Published:** 2017-12-06

**Authors:** Carolina Frankl-Vilches, Manfred Gahr

**Affiliations:** Department of Behavioural Neurobiology, Max Planck Institute for Ornithology, 82319 Seewiesen, Germany

**Keywords:** Endophenotype, Steroid receptor, Promoter, Splicing

## Abstract

Singing of songbirds is sensitive to testosterone and its androgenic and estrogenic metabolites in a species-specific way. The hormonal effects on song pattern are likely mediated by androgen receptors (AR) and estrogen receptor alpha (ERα), ligand activated transcription factors that are expressed in neurons of various areas of the songbirds’ vocal control circuit. The distribution of AR in this circuit is rather similar between species while that of ERα is species variant and concerns a key vocal control area, the HVC (proper name). We discuss the regulation of the expression of the cognate AR and ERα and putative splice variants. In particular, we suggest that transcription factor binding sites in the promoter of these receptors differ between bird species. Further, we suggest that AR- and ERα-dependent gene regulation in vocal areas differs between species due to species-specific DNA binding sites of putative target genes that are required for the transcriptional activity of the receptors. We suggest that species differences in the distribution of AR and ERα in vocal areas and in the genomic sensitivity to these receptors contribute to species-specific hormonal regulation of the song.

**Electronic supplementary material:**

The online version of this article (10.1007/s00359-017-1236-y) contains supplementary material, which is available to authorized users.

## Testosterone-sensitive singing and song pattern

Sexual behaviors, such as courtship of vertebrates in general, are linked to the reproductive cycle via steroid hormones, the androgen testosterone and the estrogen 17β-estradiol produced by the gonads. The gonadal dependency of vocal communication of male birds was known for centuries based on the castration of roosters (Berthold 1849). Data supporting the testosterone (T) dependency of vocal performance come from species of a wide variety of avian orders including galliformes species, night herons, doves, gulls, parrots, suboscine passerines and songbirds (Oscine passerines) (for review: Gahr 2014; York et al. 2016). However, individual variation in male song output and T levels are not always correlated as shown in the barn swallow (*Hirundo rustica*) (Saino and Møller 1995). Further, there are species that sing intensely even outside of the breeding season when testicles and T levels are reduced (for review Gahr 2014), continue to sing for a long time even after castration (Pröve 1974) or restart singing at the onset of the breeding season while T levels are still low (Quispe et al. 2016). Another complication for a simple relationship between T and singing activity is the fact that females of many tropical species, in particular of Australasian taxa sing regularly (Odom et al. 2014). However, there is little information about T levels of singing female birds (Geberzahn and Gahr 2011; Schwabl et al. 2015; Voigt and Gahr 2011).

In summary, pharmacological levels of T seem to stimulate singing in all cases (for review Gahr 2014); however, the link between natural levels of T and individual differences in singing behavior is unclear. There are several potential explanations for this discrepancy: first, the effect of T on song performance might involve androgenic and estrogenic metabolites of T that are produced in the brain (Schlinger and Arnold 1991). T can be converted by the enzyme 5α-reductase into the androgen 5α-dihydrotestosterone and into the 17β-estradiol via the enzyme aromatase in the brain, e.g. in male zebra finches (*Taeniopygia guttata*), estrogenic metabolites seem important for the amount of directed (presumably courtship related) singing but not for undirected singing (Walters et al. 1991). Second, T-dependent effects on behavior are generally slow processes, which can take from several days to weeks (McEwen 1994). Thus, the blood hormone concentration at the time of T’s activating or organizing activity might be very different from those sampled in parallel with the behavioral observation. Third, the definition of “high” or “low” hormone levels is likely species and sex specific, e.g., reproductively active male zebra finches have lower T levels than such male canaries (*Serinus canaria*) (Pröve 1974; Voigt and Leitner 2008), and females’ maximum levels of circulating T are in most cases lower than those of males (Ketterson et al. 2005). Fourth, due to methodological problems, hormone measurements of small animals are not possible with a high or even daily temporal resolution. Next to these problems, species, sex and individual differences in the brain expression of hormone receptors and in the regulation of hormone-receptor-mediated transcription might explain the observed heterogeneity of testosterone-sensitive singing of birds.

The song features that are sensitive to T are also highly species specific (for review, Gahr 2014): song length, song fragment length (e.g. motif, tour, phrase), song unit repertoire (element, syllable, song type), song unit stereotypy, song unit repetition rates, or the frequency range are T dependent in certain species but not in others (for review Gahr 2014). In relation, there are large species differences in the extent to which the song pattern is T sensitive, from little in the zebra finch (Pröve 1974; Arnold 1975; Walters et al. 1991; Wang et al. 2014) to stark in the canary (Heid et al. 1985; Gardner et al. 2005). Further, T and its androgenic metabolites might control vocal features that differ from the control via its estrogenic metabolites; in adult canaries, estrogens are required to sing songs with high syllable-repetition rates (Fusani et al. 2003; Rybak and Gahr 2004), a feature that is important for the sexual quality of canaries’ songs (Kreutzer and Vallet 1991). Likewise, in white-crowned sparrows (*Zonotrichia leucophrys*), androgenic and estrogenic activities in the song-control nucleus HVC mediate systemic T-dependent song stereotypy (Meitzen et al. 2007).

Although there is good experimental evidence for gonadal steroids affecting the ontogeny of singing of songbirds such as the zebra finch (Gurney and Konishi 1980) and the canary (Weichel et al. 1989), there is little developmental data that document a sex difference in ontogenetic hormone production as a possible cause for sex-specific vocal development (Hutchison et al. 1984; Schlinger and Arnold 1992; Adkins-Regan et al. 1994). This might be partially due to the technical short-comings mentioned above for the correlation of hormones and adult song.

## Hormone-dependent endophenotypes of the neural vocal control system

**Fig. 1:**
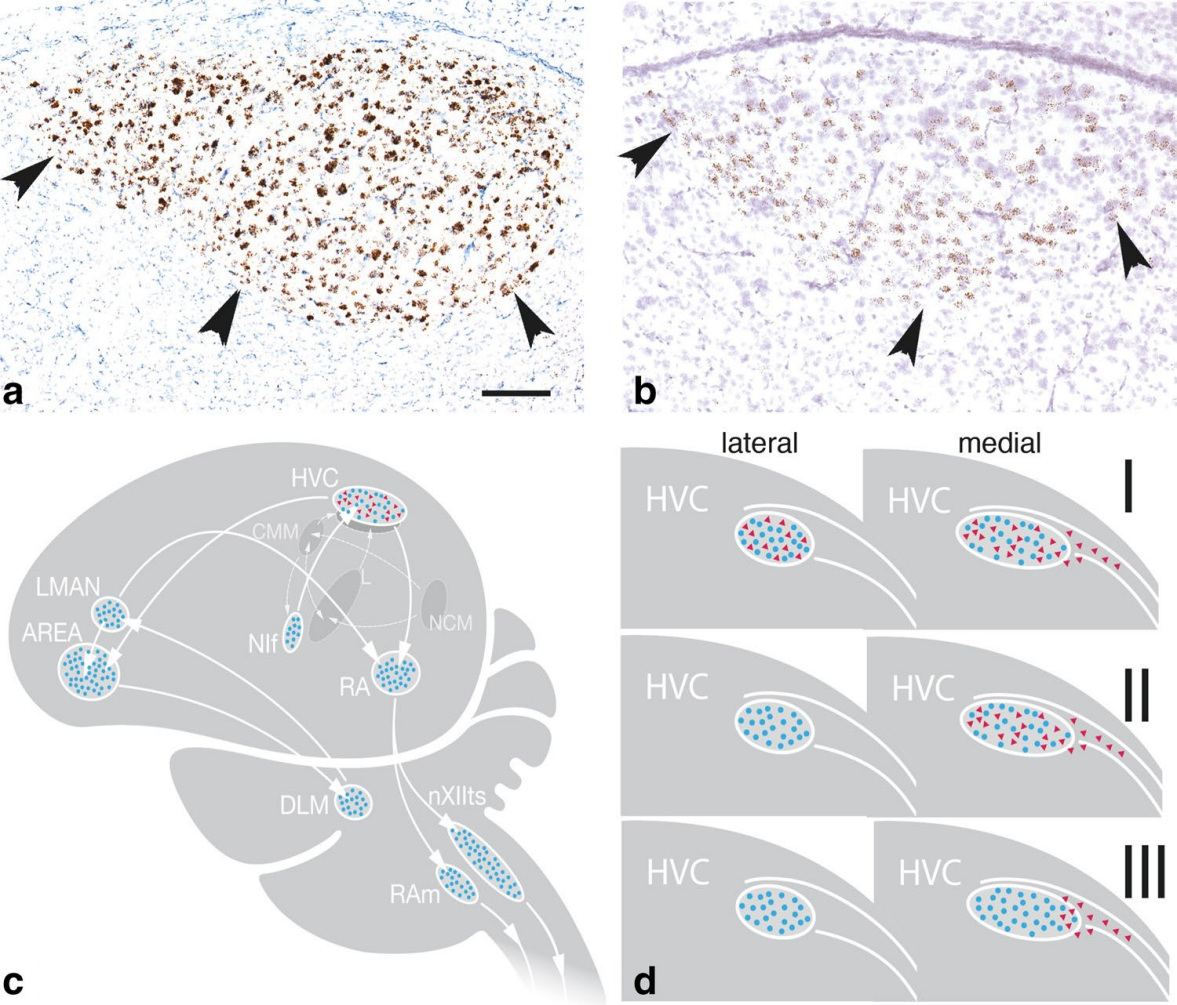
Distribution of androgen receptors (AR) and estrogen receptors (ERα) in the vocal control system. In **a**, we depict the expression of AR mRNA in the HVC of a male canary and in **b**, the ERα mRNA in the HVC of a great tit (*Parus major*) of the reproductive season. The mRNA-expressing cells (brown) were labeled with a non-radioactive in situ hybridisation method. In **c**, we show the distribution of AR (blue dots) and of ERα (red triangles) in the ares of a schematic vocal control system of songbirds. Some thalamic brain areas that appear important for coordination of the left and right vocal control network are omitted (see Wild 1997). Note that ER expression in vocal areas is limited to HVC and differs strongly between species (see **d** and Table 1). In **d**, we represent the distribution of AR and ERα in the lateral and medial part of the HVC: in type I, ERα is expressed throughout the entire HVC; in type II, ERα is expressed in the medial HVC but not or very low in the lateral part; in type III, ERα expression is low even in the medial part of HVC. In all songbirds, AR is expressed throughout HVC and ERα is found ventromedial to HVC. In Area X, AR are abundant only in some individuals. Area X; DLM, nucleus dorsolateralis anterior, pars medialis; DM, dorsomedial nucleus of the midbrain nucleus intercollicularis; HVC, proper name; Field L; lMAN, lateral magnocellular nucleus of the anterior nidopallium; mMAN, medial magnocellular nucleus of the anterior nidopallium; NC, caudal nidopallium; NIF, nucleus interfacialis; nXIIts, tracheosyringeal portion of the nucleus hypoglossus; RA, robust nucleus of the arcopallium; RAm, nucleus retroambigualis; rVRG, rostro-ventral respiratory group

The largest body of evidence of gonadal-hormone-sensitive singing (for review Gahr 2014) comes from the songbirds, which comprise about half of all living bird species. In songbirds, neural song control is achieved by a chain of interconnected brain areas in the fore-, mid-, and hindbrain (Nottebohm et al. 1976; Wild 1997; Hahnloser et al. 2002; Amador et al. 2013) (Fig. 1). In particular, forebrain vocal control areas such as the HVC (proper name) are evolutionary novelties of songbirds and involved in the learning of vocal features (Gahr 2000; Petkov and Jarvis 2012). In addition to the song pattern, these areas are active during call-based vocal communication (ter Maat et al. 2014; Benichov et al. 2016). The forebrain vocal circuit of songbirds connects to general avian vocal areas in the mid- and hindbrain via a projection of archistriatal neurons (the RA, robust nucleus of the arcopallium), in particular to the syringeal motonucleus (nucleus hypoglossus pars tracheosyringealis) and to respiratory pre-motor nuclei (Wild 1997; Wild et al. 1997).

**Table 1:** The expression of androgen receptor mRNA or protein, and estrogen receptor α mRNA or protein in the lateral and medial part of the vocal control nucleus HVC of various songbirds

Family	Species	HVC lateral	HVC medial	HVC lateral	HVC-medial
		AR	AR	ERα	ERα
Corvidae	Carrion crow (*Corvus corone*) 1	+++	++	+	++
Estrildidae	Zebra finch (*Taeniopygia guttata*) 2, 3	+++	+++	−	+
Estrildidae	Bengalese finch (*Lonchura striata dom*.) 1	+++	+++	−	+
Estrildidae	Blue-capped cordon bleu (*Uraeginthus cyanocephalus*) 1	+++	+++	−	+
Fringillidae	Canary (*Serinus canaria*) 3	+++	+++	++	+++
Fringillidae	White-crowned sparrow (*Zonotrichia leucophrys*) 3	+++	+++	+	+++
Malaconotidae	East-African shrike (*Laniarius funebris*) 4	+++	++	++	+++
Maluridae	Red-backed fairy-wren (*Malurus melanocephalus*) 1	+++	++	−	+++
Muscicapidae	Black redstart (*Phoenicurus ochruros*) 1, 5	+++	+++	−	+++
Paridae	Great tit (*Parus major*) 1	+++	++	+	++
Ploceidae	White-browed sparrow weaver (*Plocepasser mahali*) 6	+++	+++	−	++
Sturnidae	Starling (*Sturnus vulgaris*) 1, 7, 8	+++	+++	++	+++
Sylvidae	Eurasian blackcap (*Sylvia atricapilla*) 1	+++	++	+	++
Thraupidae	Silver-beaked tanager (*Ramphocelus carbo*) 1, 9	+++	+++	+	+++

Here, AR (respectively, ERα) stands for both the gene and the protein. In all cases, at least 3 males were analyzed [Data are from: 1 = Gahr unpublished; 2 = Gahr and Konishi (1988); 3 = Gahr et al. (1993); 4 = Gahr et al. (1998); 5 = Apfelbeck et al. (2013); 6 = Voigt and Gahr (2011); 7 = Balthazart et al. (1992); 8 = Bernard et al. (1999); 9 = Quispe et al. (2016). −, no labeled cells; +, low; ++, medium, +++ high number of labeled cells]

Hormone-driven song differentiation in adulthood is likely due to transient hormone-induced alterations of transcriptomes (Thompson et al. 2012; Dittrich et al. 2014; Frankl-Vilches et al. 2015) and in consequence proteomes that underlie neuroanatomical and neurophysiological endophenotypes of vocal control circuits, in particular of HVC and RA. The T-dependent neuroanatomical changes of vocal areas of adult songbirds include changes on the synaptic and dendritic level as well as changes in neuron spacing, neuron recruitment and vascularization (for review: Chen et al. 2013). Such alterations are thought to underlie seasonal hormone-dependent changes in the overall size of vocal control areas (e.g. Nottebohm 1981; Tramontin et al. 2003; but; Gahr 1990; Leitner et al. 2001); however, size measurements depend heavily on the criteria to identify vocal neurons and in consequence on the criteria to identify the boundaries of a brain area (Gahr 1997). Further, in wild canaries there are seasonal song changes despite a lack of seasonal changes of the gross morphology of vocal areas (Leitner et al. 2001), in white-crowned sparrows the size of vocal areas changes seasonally without differences in the song repertoire (Brenowitz et al. 1991). Thus, there is no simple relation between overall hormone-driven morphological changes and hormone-dependent song patterns of songbirds. This conclusion might also be affected by species differences in the hormone sensitivity of the song pattern and of the endophenotypes of the vocal areas.

To understand how various gonadal hormone-dependent neural properties relate to vocalization in various species requires electrophysiological and genetic approaches. T-treatment of adult male white-crowned sparrows (simulating breeding conditions) showed that membrane capacitance, evoked and spontaneous firing rates of RA projection neurons increased, while the electrophysiological properties of HVC interneurons and projection neurons remained stable (Meitzen et al. 2009). Intracerebral hormonal manipulations of these birds showed that the effects of systemic T on RA neurons are mediated via androgenic and estrogenic activity within HVC, but not in RA (Meitzen et al. 2007). In contrast, in adult zebra finches, T did neither affect synaptic transmission nor dendritic length and spine density of RA neurons but affected these parameters of lMAN (lateral magnocellular nucleus of the anterior nidopallium) (White et al. 1999). Castration of adult zebra finches reduced the excitability of RA neurons that project to the brainstem (Wang et al. 2014). In summary, exogenous T can have selective actions on different vocal control areas and neuron populations and these actions might differ between species. One possible explanation of these differences would be the differential expression of hormone receptors in the various vocal areas, neuron populations and species (see below).

## Modes of steroid action

One mode of steroid action in the brain is the alteration of gene expression by binding to intracellular steroid receptors that transactivate transcription of target genes in a ligand-dependent manner (Carson-Jurica et al. 1990). The androgen receptor (AR) has a high affinity for the androgens T and 5α-dihydrotestosterone, but not for 5β-dihydrotestosterone (Grino et al. 1990). The two types of estrogen receptors (ERα, ERβ) bind 17β-estradiol with high affinity. The AR gene codes for the AR protein, the ESR1 gene for ERα, and the ESR2 gene for ERβ. Coactivators of the receptor complex are important for the specificity and/or affinity of the receptor for their cognate ligand and for receptor–DNA binding (Yeh et al. 1998; Nilsson et al. 2001). In rodents and humans, ESR1 and AR mRNAs display high heterogeneity due to alternative splicing, which might lead to truncated variant proteins with either ligand-independent constitutive actions or unknown functions (Stellato et al. 2016; Xu and Qiu 2016; Ishii et al. 2017).

A second mode of androgen and estrogen action is the direct rapid alteration of neuronal properties and brain functions (non-genomic mechanism) (Pouliot et al. 1996; Moss and Gu 1999; Sellers et al. 2015) via special membrane receptors such as GPER1/GPR30 (Hadjimarkou and Vasudevan 2017) and ZIP9 (Thomas et al. 2017), and classical ERs and ARs located in extra-nuclear compartments that might be linked to second messenger pathways (Nilsson et al. 2001; Lucas-Herald et al. 2017; Hadjimarkou and Vasudevan 2017).

The slow onset of most androgen- and estrogen-dependent development and induction of song behaviors are characteristic of transcription- and translation-based activity of steroids. Since classical AR and ERα (see review below) are expressed in neurons of vocal control areas, in this review we focus on the structure, regulation and neural distribution of AR and ERα as well as the sensitivity of the birds’ genomes to these receptors in relation to the hormone sensitivity of singing.

## Androgen and estrogen receptors in the vocal control system

Comparative studies of the distribution of ERα- and AR-expressing cells in vertebrate brains showed that the brain regions, which typically contain such cells, are evolutionarily conserved among vertebrates (e.g. hypothalamic-preoptic areas and the amygdala) or are linked to taxa-specific sexual behaviors (Pfaff 1980; Kim et al. 1978). ERα- and AR-expressing areas, such as the medial preoptic area and the medial amygdala, are likely required for hormone-dependent singing activity of birds (Hutchison and Steimer 1984; Alward et al. 2013; Horton et al. 2014; Cordes et al. 2015). Vocal control areas of songbirds are an example of taxa-specific AR- and ERα-expressing neurons (Balthazart et al. 1992; Gahr et al. 1993; Metzdorf et al. 1999; Bernard et al. 1999; Gahr 2000).

In songbirds, AR mRNA or AR protein were reported for HVC, RA, and lMAN of all species studied (Balthazart et al. 1992; Bernard et al. 1999; Gahr et al. 1998, 2008; Metzdorf et al. 1999; Fusani et al. 2000; Voigt and Gahr 2011; Fraley et al. 2010; Quispe et al. 2016). Since these include species of various songbird families, among which are the basal Maluridae, the Corvidae, the Malaconotidae, and the derived Fringillidae and Thraupidae, the AR expression in HVC, RA and lMAN seems a general characteristic of songbirds (Fig. 1; Table 1; see Barker et al. 2004 for Systematik of Songbirds). Further, AR mRNA and protein are reported for mMAN (medial magnocellular nucleus of the anterior nidopallium) and NIF (nucleus interfacialis) in canaries and zebra finches (Balthazart et al. 1992; Metzdorf et al. 1999; Fusani et al. 2000), but these areas have not yet been surveyed in other species. Nevertheless, extrapolating from the HVC, RA and lMAN data, we assume that AR expression in mMAN and NIF is also a common feature of songbirds. In Area X of zebra finches and canaries, ARs occur in only some individuals for unknown reasons (Gahr 2004; Kim et al. 2004). In another Estrildid finch, the wild white-rumped munia (*Lonchura striata*) and its domesticated relative the Bengalese finch (*Lonchura striata dom*.), ARs are expressed in a strain-specific pattern in Area X (Wada et al. 2013).

Although there might be individual differences in the expression of ARs in vocal control areas, the expression pattern between species is very similar and does not explain species differences in the degree of T-sensitivity of song features. Such correlation might require detailed coexpression studies of ARs and neuron type-specific markers. Nevertheless, species differences in seasonal dynamics of AR expression in vocal control areas might be involved in seasonality of song pattern and neural endophenotypes in a species-specific way (Fusani et al. 2000; Fraley et al. 2010).

Among forebrain vocal areas, ESR1 mRNA and ERα protein is only expressed in HVC and around the dorsal aspect of RA of canaries and zebra finches (Gahr et al. 1993; Metzdorf et al. 1999) while ERβ mRNA is not expressed in any of the vocal areas (Bernard et al. 1999). Further comparative data are available for ERα expression and protein abundance in the HVC of various species (Table 1). These data suggest three types of distribution pattern (Fig. 1d; Table 1): (1) high expression of ERα throughout the entire HVC (e.g. canary, East-African shrike); (2) high expression of ERα only in the medial part of HVC (e.g., the forest weaver, the black redstart); (3) no expression in the lateral part and low levels of ERα in the medial part of HVC (e.g. zebra finch, Bengalese finch). More species are needed to classify these species differences as species, genus or family-typical pattern. In all songbird species, a large population of ERα-expressing neurons is found ventromedial to HVC aligning the lateral ventricle, an area including the so-called para-HVC (Johnson and Bottjer 1995), but extending much further medial than the latter (Gahr et al. 1993, and unpublished data).

Next to the forebrain vocal control areas, ARs and ERαs are expressed in sub-areas of the caudal nidopallium (Gahr et al. 1993; Metzdorf et al. 1999) that are indirectly connected to the vocal control system of the zebra finch and canary (Bolhuis and Gahr 2006). The lack of comparative data does currently not allow generalization of these observations to other songbird species. In the brainstem, ARs occur in all respiratory–vocal areas and in syringeal motoneurons (Gahr and Wild 1997; Gahr 2000).

During development, AR mRNA was first detected in RA around posthatching day 5 and in HVC at posthatching day 9 (Gahr and Metzdorf 1999; Perlman et al. 2003; Kim et al. 2004). The nucleus hypoglossus and the syrinx express AR mRNA in male and female embryos of zebra finches (Godsave et al. 2002). ESR1 mRNA appears first in the caudal nidopallium of male and female zebra finches in and close to HVC in the first 2 weeks of post-hatching life (Gahr 1996; Jacobs et al. 1999). At 30 days of age, neurons of the entire HVC expressed ERα in canaries while ERα was only found in the lateral HVC of zebra finches (Gahr and Konishi 1988; Gahr et al. 1996; Gahr 1996). Thus, the AR and ERα distributions of adults described above are rather similar to those of juveniles, although zebra finches lose much of their ERα expression in the medial HVC during development, i.e. develop from type II to type III (Gahr and Konishi 1988). Likewise, AR expression might change somewhat in various vocal areas during ontogeny as suggested by androgen accumulation studies (Bottjer 1987).

Since the same vocal control areas contain AR and ERα mRNA and protein in male and female songbirds (Gahr and Konishi 1988; Gahr et al. 1993, 1996; Metzdorf et al. 1999; Gahr and Metzdorf 1999; Jacobs et al. 1999; Kim et al. 2004) sex steroids can directly affect the vocal control areas and vocal phenotypes in both males and females.

## Receptor structure: species differences and tissue-specific splice variants

There are large differences in the neural distribution of AR- and ERα-containing neurons between avian orders and within the passeriformes, between the oscine and sub-oscine suborders (Gahr et al. 1993; Gahr 2000). Species differences in the area-specific expression are likely due to species differences in either the promoter structure of steroid receptors, and/or the local availability of relevant transcription factors that control the expression of AR and ERα. In addition, sex, developmental, and individual differences might involve epigenetic modification of the promoters of AR and ERα, splice variants and nucleotide polymorphisms of the receptors, as well as differences in circulating androgens and estrogens, in light of the autologous and heterologous regulation of ERα and AR shown for rodents (Burgess and Handa 1993; Lisciotto and Morell 1993). Likewise, T had a short-term inhibitory effect on the expression level of AR mRNA in HVC of canaries but long-term treatment did not affect AR mRNA levels (Nastiuk and Clayton 1995; Fusani et al. 2003). In the following we discuss the gene structure of AR and ESR1 and its promoters.

### Alternative splicing and nucleotide polymorphisms

**Fig. 2:**
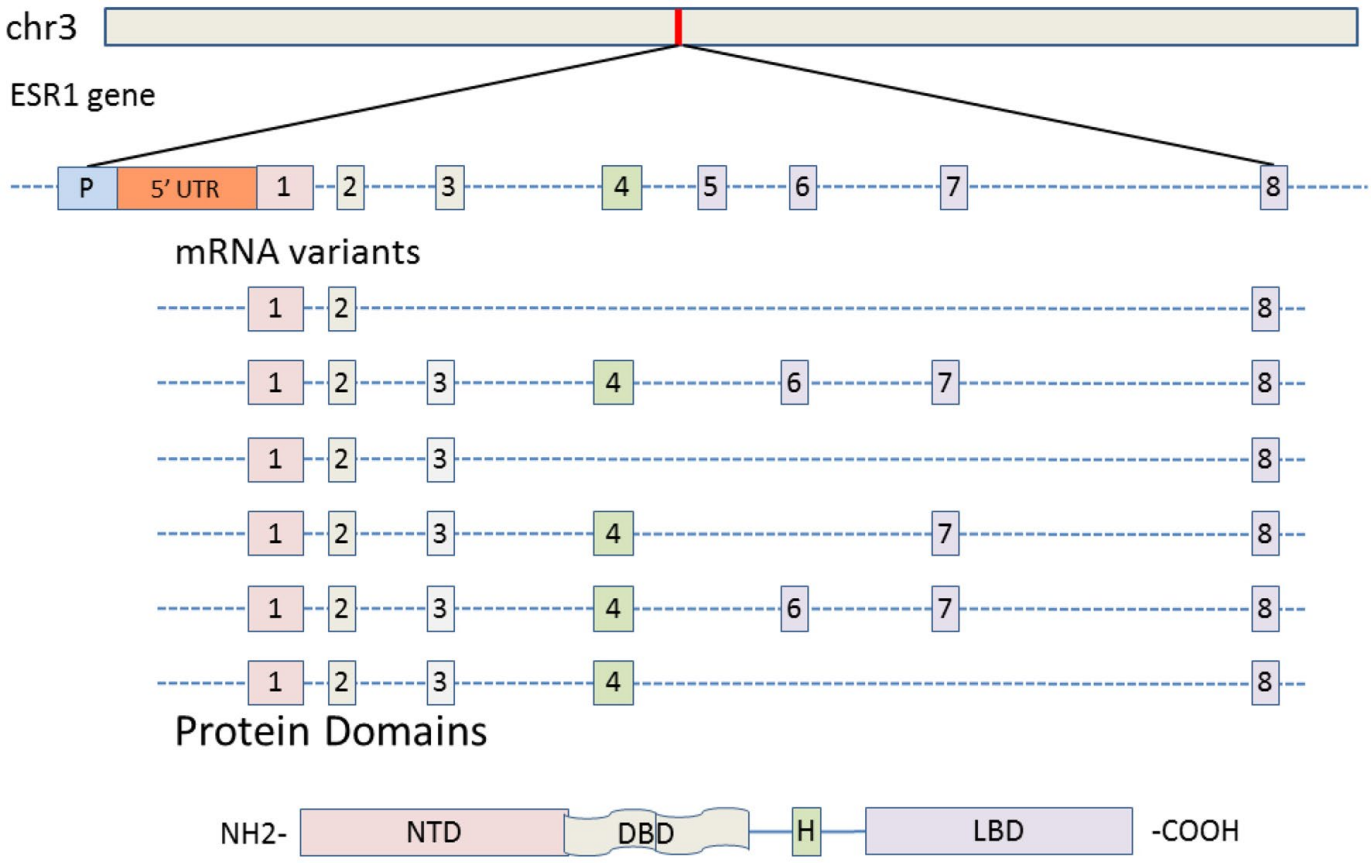
Estrogen receptor gene (ESR1) structure and alternative splice variants. The eight exons are color-coded relative to the encoded protein domains of the ERα. Splice variants were found in the hypothalamus of the zebra finch. In most variants, the hinge region (H) and the ligand binding domain (LBD) were missing. *DBD* DNA binding domain, *NTD* N-terminal domain

**Fig. 3:**
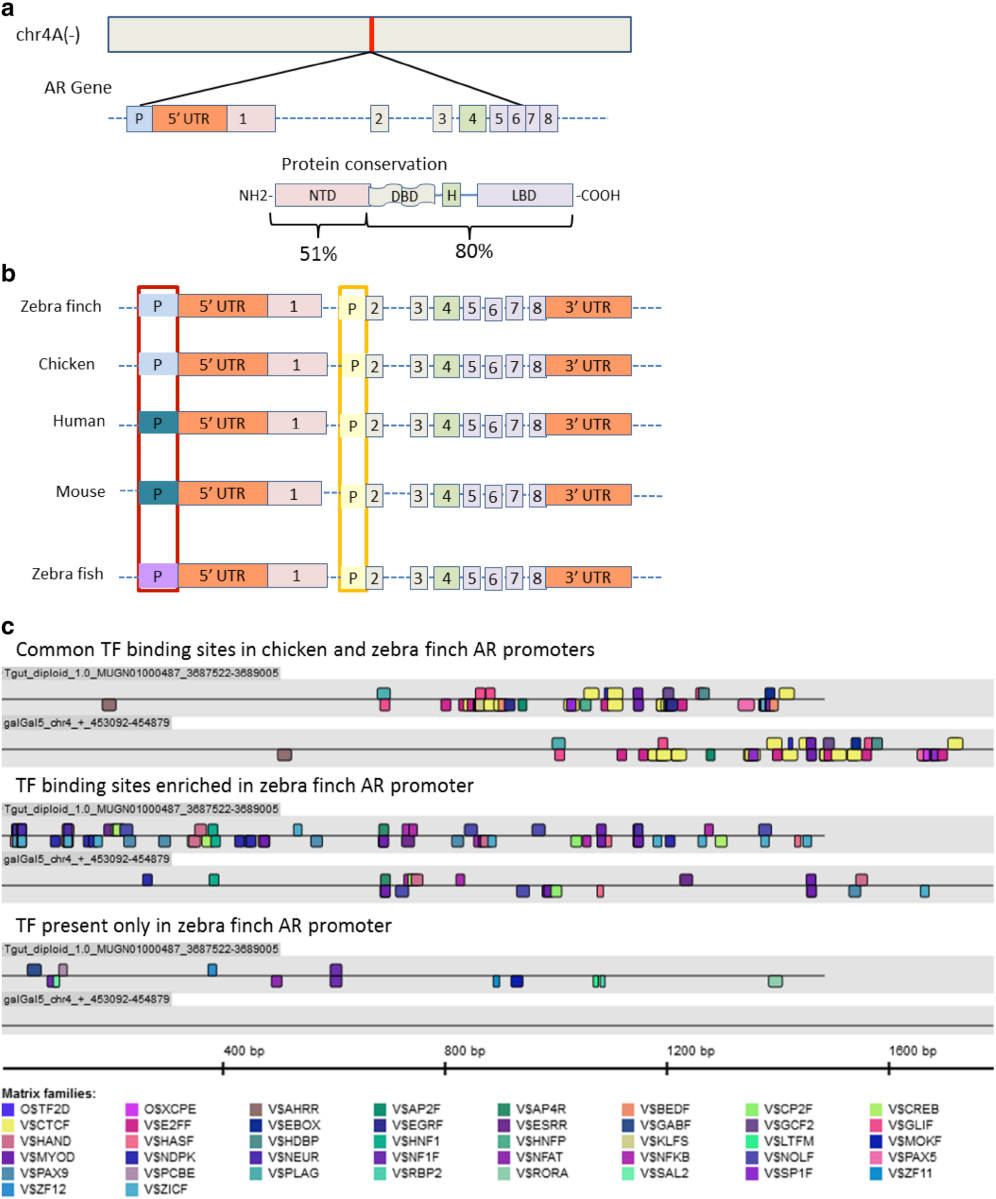
**a** The androgen receptor (AR) gene is composed of eight coding exons: exon 1 encodes the amino terminal domain (NTD, pink), exons 2 and 3 encode the DNA binding domain (DBD, gray), exon 4 encodes the hinge region (H, green), exons 5–8 encode the ligand binding domain (LBD, violet). The DBD, H, and LBD are highly conserved among vertebrates, whereas NTD is less conserved (protein conservation scores are based on Constraint-based Multiple Alignment Tool available by NCBI; https://blast.ncbi.nlm.nih.gov/Blast.cgi). **b** Comparative genomic analysis of putative promoters of the AR in vertebrates. The analysis identified two sets of putative promoters in avian AR genes. One set is placed in front of exon 2 (yellow). This sequence was highly conserved among vertebrates (yellow box), and a second promoter in front of the 5′ flanking exon 1 region (red box), which is class specific, i.e. differed between birds (blue), mammals (petroleum) and fish (purple). The promoter differences were based on sequence similarity scores (see Supplementary Table 1). The annotation of the zebra finch AR gene and its orthologues was done with ElDorado (genomatix genome annotation of publically available reference genomes). **c** Common, species-enriched, and species-specific transcription factor (TF) binding motifs in the exon 1 promoter of the AR shown in **b** (blue). Transcription factor binding sites were identified in silico with MatIsnpector and FrameWorker (Genomatix GmbH). AR gene sequences of the zebra finch (PacBio data of TGU_diploid_1.0; GCA_002008985.2) and chicken (galGal5 (GCA_000002315.3)) were analyzed by PromoterInspector (Genomatix GmbH) to predict eukaryotic polymerase II binding in genomic sequences (Scherf et al. 2000). The scaffold/chromosome allocation and coordinates of the promoter predictions are shown

AR and ESR1 evolved from ancient receptors by two large-scale genome expansions, one before the advent of jawed vertebrates and one after (Thornton 2001). Both receptors are composed of eight protein-coding exons (Figs. 2, 3a). In particular, exons encoding for the DNA binding domain (DBD), the hinge region (H) and the ligand binding domain (LBD) are highly conserved even between mammals and birds while the amino terminal domain (NTD) is less conserved in vertebrates (Fig. 3a). Further, both AR and ERα have variable numbers of untranslated exons (5′ UTRs) and as such might have additional promoters next to the promoter adjacent to the transcription start site.

Splice variants of the AR and ESR1 are frequently found in prostata (Wadosky and Koochekpour 2016; Karantanos et al. 2015) and breast cancer (Hu et al. 2014; Forootan et al. 2016), but are as well found in the brain of mammals including humans (Hu et al. 2014; Ishunina et al. 2013; Kundu et al. 2015). AR splice variants have not been analyzed in birds. In zebra finches, we found several ESR1 splice variants (Fig. 2) occurring in higher density in neurons distributed throughout the hypothalamus (Gahr and Metzdorf unpublished data), hence a functional role is likely. Most of these variant proteins would preserve the DNA binding domain, i.e., would have none or only a truncated ligand-binding domain. C-terminally truncated ERα (or AR) proteins potentially display ligand-independent transcriptional activity (Stellato et al. 2016; Xu and Qiu 2016; Ishii et al. 2017). Alternatively, additional functions for truncated receptor proteins could be to act as competitive antagonist, to impair receptor transactivation, and to inhibit protein– and/or DNA–protein interaction, thus modulating the activity of full-length receptors (Monaghan and McEwan 2016). The biological relevance of such AR and ERα variants in the bird brain for the hormonal control of song and the vocal control system differentiation needs to be evaluated.

Nucleotide polymorphisms of AR and ESR1 of mammals have been studied in great detail, in particular in relation with carcinoma differentiation (Dos Santos et al. 2017; Eisenegger et al. 2017) and are thought to correlate with human behavioral and neurological problems (Maney 2017). In birds, such analysis in association with neural and behavioral phenotypes is missing.

### Species-specific promoters of the androgen receptor (AR)

A possibility for order and species differences in the neural distribution of AR and ERα are differences in promoter structure of the AR and ESR1 gene. Since high-quality genomes (produced by PacBio sequencing) are available for chicken and zebra finches, we compared the promoter structures of the AR gene of these species and those of various mammals and the zebra fish (Fig. 3, Suppl. Table 1). The gene body sequences were analyzed by PromoterInspector (Genomatix GmbH), which predicts eukaryotic polymerase II promoter regions with high specificity in genomic sequences (Scherf et al. 2000). For the ESR1 of birds we expect a similar result.

The AR of birds seems to have one promoter that is similar to those of mammalian AR (in front of exon 2) and a second species-specific AR promoter in the 5′ UTR in front of exon 1 (Annotation El Dorado, Genomatic GmbH) (Fig. 3b). The general promoter had very similar sequences in all species while that in front of exon 1 were most similar between species of the same vertebrate class, i.e. between birds and between mammals, respectively (see Supplementary Table 1 for similarity data). The exon 1 promoter is identical to the human minimal promoter of the AR (Takane and McPhaul 1996). We do not know yet, whether the putative general promoter in front of exon 2 of all analyzed species is used for transcription of AR variants.

The promoter sequences of the species/class-specific promoters were extracted and analyzed for transcription binding sites by MatInspector (Genomatix GmbH, http://www.genomatix.de) (Cartharius et al. 2005). When comparing putative transcription factor binding sites, so-called motifs, of zebra finches and chicken, we found common binding motifs, enriched motifs (i.e. several motifs of the same type) in one species, and species-specific motifs; we show the common, the zebra finch-enriched and the zebra finch-specific motifs (Fig. 3c). These comparisons show clear species differences in putative transcription factor binding sites next to similarities between zebra finch and chicken. Regarding the putative zebra finch-specific motifs, we can just speculate about the role of various transcription factors for the regulation of AR. In studies of castration-resistant prostate cancer the AR expression can be directly modulated by the retinoic acid receptor-related orphan receptor gamma (ROR-γ) (Wang et al. 2016), a member of the V$RORA family. It shall be interesting to see if bird species within the same family (e.g. zebra finch and Bengalese finch) have more conserved transcription factor motifs in their AR promoters than species as distant as zebra finch and chicken that diverged about 70 million years ago.

Such species differences of the binding motifs of the AR and ESR1 might be functionally meaningful in the bird brain was suggested for the singing behavior of the white-throated sparrow (*Zonotrichia albicollis*) (Horton et al. 2014). These authors report a strain-specific difference in the ESR1 promoter that correlates with a higher expression of ESR1 mRNA in the medial amygdala of the white-striped morph, showing a higher singing activity as well as more aggressive behavior. However, it needs to be noted that these strains differ in a large chromosomal rearrangement that includes about 1000 genes next to the ESR1 (Maney et al. 2015).

Despite this promising finding in the white-throated sparrow, generally it is unclear how species differences in area-specific expression of AR and ESR1 in the brain are controlled. It still needs to be seen whether the above-described differences in AR promoter sequences are important for the differences in AR distribution in the brain of zebra finches and chicken (Gahr 2000). Critically, the regulation of AR and ESR1 expression is highly complex and varies considerably in different tissues, and cell types even within a species due to their multiple promoters, to the epigenetic regulation of the promoters, and to multiple transcription factors that can activate AR and ESR1 expression, in part dependent on the presence of coactivators (Imamura 2011; Matsuda 2014; Wang et al. 2016). Precise epigenetic regulation of AR and ESR1 in relation to the development of sexual hormone-dependent brain areas and behaviors has been studied in mammals (for review: Matsuda 2014), but not in relation to birdsong.

## Hormone-responsivity of genes and the genome

The genomes of songbirds contain about 17,500–16,300 protein coding genes (e.g. Warren et al. 2010; Frankl-Vilches et al. 2015). The discrepancies in the number of such genes between published songbird genomes (Poelstra et al. 2014; Qu et al. 2013; Warren et al. 2010; Frankl-Vilches et al. 2015), likely, reflect technical shortcomings of the various sequencing and assembly approaches. In the canary genome, Frankl-Vilches and colleagues (Frankl-Vilches et al. 2015) failed to find the duplications (caspase 3, beta secretase, growth hormone) and large expansions of gene families (PAK3, PHF7, PIM1L) coding for brain-expressed proteins that were previously reported for the zebra finch (Warren et al. 2010; Lovell et al. 2014). These findings suggest that the evolution of hormone-sensitive singing and related endophenotypes of songbirds results from gain and loss of genes (Lovell et al. 2014), as well as from the hormone-sensitive differential regulation of genes that exist in all songbird genomes (Frankl-Vilches et al. 2015). Similar conclusions have been drawn in great apes, where the genes of chimpanzees and other apes differ only marginally from those of humans (Prufer et al. 2012), even though only the latter possess speech capabilities.

In contrast to the global similarity of songbird genomes, on the nucleotide level there are considerable species differences as shown above for the AR promoter of chicken and zebra finch (Fig. 3c; Suppl. Table 1). Such species differences can impact binding motifs of the ERα, the so-called estrogen response element (ERE) and of the AR, the so-called androgen response element (ARE) as shown for genes expressed in the HVC of the canary and the zebra finch (Frankl-Vilches et al. 2015). The canonical ERE and ARE, respectively, are two hexameric half-sites of a consensus nucleotide sequence, arranged as inverted repeats, separated by three spacer nucleotides; sequences are different between ERE and ARE and several related elements such as half-site ARE and ERE exist (Klein-Hitpass et al. 1986; Claessens et al. 1989) to which the receptors have lower affinity.

**Fig. 4:**
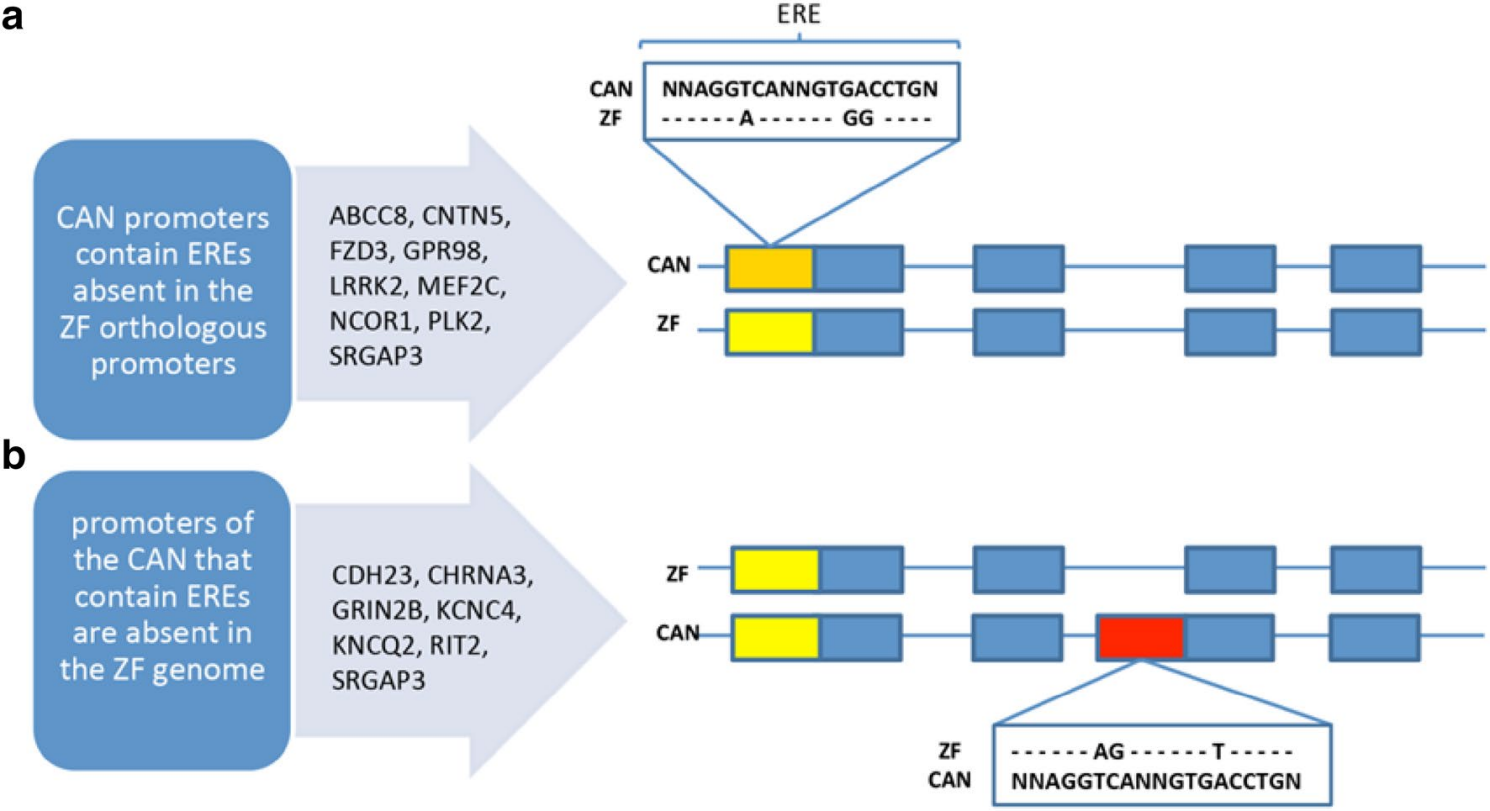
Canary-specific estrogen response elements (EREs, see insert) in orthologous genes of canary and zebra finch. In **a**, we list genes of which we found the orthologous promoters but that contain nucleotide sequences in the zebra finch deviating from known ERE sequences. In **b**, we list genes with EREs in the promoters of canaries of which we did not find the orthologous promoters of zebra finches. In these cases, sequence analysis of 1 kb of the putative promoter region of the zebra finch did not identify EREs. The 15 genes analyzed in detail were randomly selected from the list of genes with canary specific EREs. Of about 550 genes that were seasonally upregulated in HVC and that contain canary specific EREs, about one-half are classified as type “a” and the other half as type “b”. Some genes such as SRGAP3 are in both categories. Orthologous promoters are in yellow, those containing EREs are in orange, and heterologous promoters are in red (from Frankl-Vilches et al. 2015)

We studied putative AREs and EREs within 1 kb of transcription start sites of genes of canaries that were T-responsive in the HVC of canaries (Frankl-Vilches et al. 2015). Since seasonal- or testosterone-responsive genes of canaries on average contain 2–4 AREs or EREs, the evolution of such sites of a gene requires several point-mutations or larger genome modifications such as inventions of entire promoters. There was an important species-difference in the potential hormone sensitivity of these genes: About 35% of the ERE- and about 11% of ARE-bearing genes expressed in HVC of canaries were lacking these sites in the corresponding zebra finch orthologous promoters (Fig. 4) (Frankl-Vilches et al. 2015). Because only canonical ARE and ERE and only within 1 kb of transcription start sites of testosterone-sensitive genes in the canary HVC were considered (Frankl-Vilches et al. 2015), the difference in the number of genes that have functional hormone binding sites in the canary and zebra finch genome may be larger than what we report here. The number of AREs and EREs is increasing dramatically with increased distance to the transcription start site as shown in mammals (Lin et al. 2007; Stender et al. 2010; Hu et al. 2010). By means of bioinformatics, 70.000 putative EREs have been identified in the human genome, about 17,000 within 15 kb of transcription start sites of genes (Bourdeau et al. 2004).

The canary-specific evolutionary loss or gain (e.g. through point mutations) of EREs and AREs leads to species-specific gene pools that can be regulated by the activation of AR and ERα via T and its androgenic and estrogenic metabolites in HVC. Thus, AR and ERα could regulate transcription in the canary HVC or other brain areas due to the evolution of species-specific hormone-responsive *cis*-regulatory sites. The putative androgen- and/or estrogen-sensitive sites of the genome are only partially conserved even between relatively closely related songbird species, similar to mammals (Lin et al. 2007; Hu et al. 2010). e.g., only 62% of the ARE motifs identified with ChIP-seq in mouse epididymis tissue are conserved in the rat (Hu et al. 2010). This suggests that conclusions regarding androgen- or estrogen-sensitive gene networks and functions, such as HVC transcriptomes and singing, of any particular songbird species might be highly species-specific and requires genomic information from that species.

## Summary

The expression of AR in vocal control areas of songbirds is rather similar between species while the presence of ERα in an important such area, the HVC varies between species. However, more comparative receptor expression studies are required to link such species differences to species differences in hormone-sensitive singing and to correct for phylogenetic confounds. Likewise, further high quality genomes of species with known hormone-sensitivity of song pattern are needed to confirm that species differences of genomic AR and ERα binding sites are an important regulatory mechanism for species-specific behavioral pattern. Last, the role of splice variants of AR and ERα in neuronal and neural regulatory mechanism of birds and vertebrates in general is little understood. In summary, we suggest that next to the production of the androgens and estrogens per se, there are different regulatory levels of hormone-sensitive singing: (1) the presence/absence of functional AR and ERα, (2) the species-specific enrichment of the DNA hormone-responsive elements.

## Electronic supplementary material

Below is the link to the electronic supplementary material.

Supplementary material 1 (DOCX 104 KB)

## Abbreviations

AR: Androgen receptor
ARE: Androgen response element
Area X: Vocal control area (proper name)
ERα: Estrogen receptor alpha
ERβ: Estrogen receptor beta
ERE: Estrogen response element
ESR1: Estrogen receptor alpha gene
ESR2: Estrogen receptor beta gene
HVC: Vocal control area (proper name)
lMAN: Lateral magnocellular nucleus of the anterior nidopallium
mMAN: Medial magnocellular nucleus of the anterior nidopallium
NIF: Nucleus interfacialis
RA: Robust nucleus of the arcopallium
T: Testosterone
