# Supplementary material for: Androgen and estrogen sensitivity of bird song: a comparative view on gene regulatory levels
Source: J Comp Physiol A Neuroethol Sens Neural Behav Physiol. 2017 Dec 6;204(1):113–26. doi: 10.1007/s00359-017-1236-y (PMC5790841; doi:10.1007/s00359-017-1236-y)
Supplement: Supplementary file 1 — Supplementary material 1 (DOCX 104 KB) [file 359_2017_1236_MOESM1_ESM.docx]

**Supplementary Information**

**
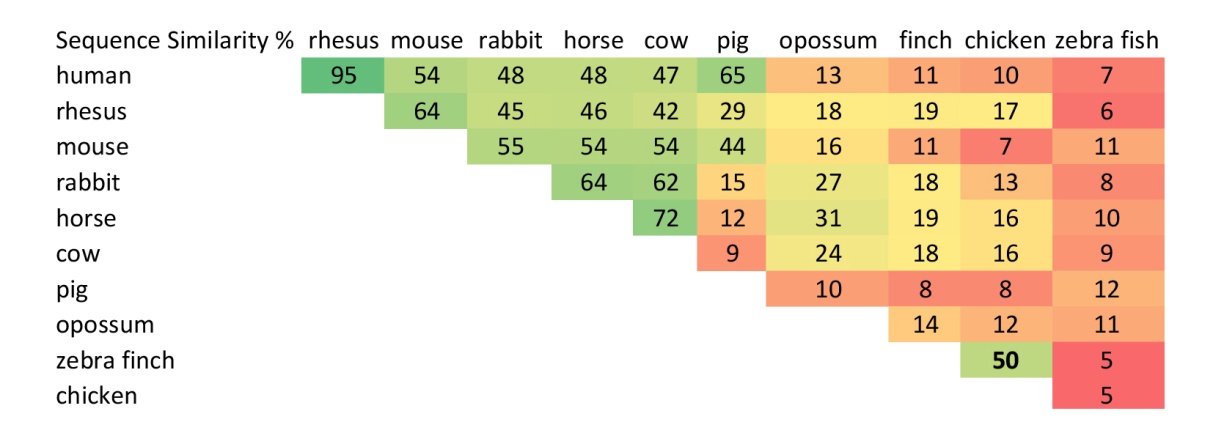
**

**Supplementary table 1.**

Similarity of the androgen receptor exon 1 promoter sequences of birds, mammals and the zebra fish. Pairwise similarity scores are shown in percentage based on the number of identical nucleotides of AR promoter sequences for full length RNAs among vertebrates. The zebra finch is most similar to the chicken, and eutherian mammals are most similar to other eutherian species but different from the opossum. Sequences were extracted from the available genome annotations of ElDorado (Genomatix GmbH). Abbreviations: rhesus = rhesus monkey; finch = zebra finch.
